# Novel association between atherogenic index of plasma and bone mineral density in men: a retrospective analysis

**DOI:** 10.1186/s12902-025-02128-3

**Published:** 2026-01-06

**Authors:** Bing Liu, Yue Liu, Qing Xue, Fei Gao, Hao Qi

**Affiliations:** 1 Department of Endocrinology, First Hospital of Shanxi Medical University, Taiyuan, Shanxi Province China; 2 Department of Nuclear Medicine, First Hospital of Shanxi Medical University, Taiyuan, Shanxi Province China; 3 The First Clinical Medical College of Shanxi Medical University, Shanxi Medical University, Taiyuan, Shanxi Province China; 4 China Academy of Medical Sciences, Shanxi Medical University, Taiyuan, Shanxi 030000 China

**Keywords:** Male osteoporosis, Atherogenic index of plasma, Lipid profiles, Bone mineral density, HDL

## Abstract

**Background:**

This study aimed to investigate the association between the atherogenic index of plasma (AIP), lipid parameters, and bone mineral density (BMD) in men with osteoporosis.

**Methods:**

This retrospective, single-center study included 430 male patients aged ≥ 50 years admitted to the First Hospital of Shanxi Medical University between June 2022 and December 2024. Routine blood lipid profiles, including total cholesterol (TC), triglycerides (TG), high-density lipoprotein cholesterol (HDL), and low-density lipoprotein cholesterol (LDL) were collected. AIP was calculated as log10(TG/HDL). BMD was measured using dual-energy X-ray absorptiometry (DXA) and patients were categorized into normal (NO), osteopenia (ON), and osteoporosis (OP) groups. Correlation analysis, principal component analysis (PCA), and one-way Analysis of Variance (ANOVA) were applied.

**Results:**

Among 430 participants, 218 were classified as NO, 133 as ON, and 79 as OP. PCA revealed that the first two components (PC1 eigenvalue = 2.127, PC2 eigenvalue = 1.531) explained 73.15% of the total variance (PC1: 42.53%, PC2: 30.62%), with AIP and TC identified as major contributors to BMD variation. Correlation analysis showed that AIP (*r* = -0.26,*p* = 0.003), TG (*r* = -0.15,*p* = 0.047), and HDL (*r* = 0.34,*p* < 0.001) were significantly associated with BMD, while TC and LDL were not. ANOVA demonstrated significant group differences in AIP and HDL, particularly between the OP and NO groups.

**Conclusion:**

Elevated AIP and altered HDL levels are associated with reduced BMD in men, highlighting their potential role in the early identification and risk stratification of male osteoporosis.

**Supplementary Information:**

The online version contains supplementary material available at 10.1186/s12902-025-02128-3.

## Mini abstract

This study examined the association between blood lipids and bone mineral density (BMD) in men. Among 430 participants, BMD showed significant correlations with the atherogenic index of plasma (AIP, *p* = 0.003), triglycerides (TG, *p* = 0.047), and high-density lipoprotein cholesterol (HDL, *p* < 0.001). Elevated AIP and altered HDL levels were linked to lower BMD, suggesting these lipid markers may serve as cost-effective indicators for assessing osteoporosis risk in men.

**Supplementary Information:**

The online version contains supplementary material available at 10.1186/s12902-025-02128-3.

## Introduction

Osteoporosis is a systemic skeletal disorder characterized by reduced bone mass and microarchitectural deterioration, leading to increased bone fragility and fracture risk [1, 2]. It is generally classified into two types: primary and secondary osteoporosis [3].Primary osteoporosis develops in the absence of other diseases, most commonly in postmenopausal women due to estrogen deficiency, and in elderly individuals as a consequence of age-related bone loss [4].

Although osteoporosis is often perceived as a women’s disease, it also represents a major health concern in men. Approximately 20% of the 44 million Americans with osteoporosis or low bone density are men, who account for 30–40% of osteoporotic fractures. The lifetime fracture risk among men aged ≥ 50 years is estimated to range from 13% to 30% [5, 6].Secondary osteoporosis, by contrast, arises from identifiable causes such as endocrine disorders (e.g., hyperthyroidism, hypercortisolism), chronic diseases (e.g., diabetes, chronic kidney disease, inflammatory bowel disease), or long-term glucocorticoid therapy [7, 8].Epidemiological data suggest that secondary osteoporosis affects up to 30% of postmenopausal women, more than 50% of premenopausal women, and 50–80% of men [7]. These observations highlight the substantial clinical and public health burden of osteoporosis in both sexes and underscore the importance of improved risk stratification and preventive strategies.

Certain underlying diseases associated with secondary osteoporosis, such as diabetes and chronic kidney disease, can alter lipid metabolism, contributing to bone loss. Diabetic patients often have dyslipidemia, with elevated triglycerides and reduced HDL, promoting inflammation and oxidative stress that worsen bone resorption [9]. Similarly, chronic kidney disease leads to lipid disturbances, including increased LDL, further impairing bone metabolism [10]. Understanding these lipid abnormalities can help identify high-risk individuals and guide prevention efforts.

This link between lipid metabolism and bone health is not only relevant in the context of secondary osteoporosis but also in primary osteoporosis.In recent years, AIP, defined as log10(TG/HDL), has emerged as a strong predictor of cardiovascular disease [11–14]. AIP is associated with small dense LDL particles and increased cholesterol esterification rates, reflecting adverse lipid metabolism. Beyond its role in cardiovascular disease, AIP has been linked to trabecular bone microarchitecture deterioration [15], suggesting a potential interplay between lipid and bone metabolism.

Recent studies have further demonstrated that elevated AIP levels are independently associated with reduced bone mineral density and increased fracture risk in various populations, including postmenopausal women and individuals with metabolic disorders [15, 16]. Moreover, AIP has been linked to markers of oxidative stress and inflammation that promote osteoclast activation and impair osteoblast differentiation, suggesting a potential mechanistic pathway between dyslipidemia and bone loss [17, 18].

Although research on AIP and osteoporosis has primarily focused on postmenopausal women [16], its relationship with bone health in men remains largely unexplored. Given the growing recognition of AIP as an indicator of bone quality and turnover, investigating this association in men could yield valuable insights into sex-specific pathophysiological mechanisms. Therefore, this study aimed to elucidate the associations between AIP, lipid parameters, and BMD in men, and to evaluate their potential predictive value for the early detection of osteoporosis.

## Data and methods

### Study population

Male patients aged ≥ 50 years admitted to the Department of Endocrinology, First Hospital of Shanxi Medical University, between June 2022 and December 2024 were retrospectively included.

### Inclusion criteria and exclusion criteria

Inclusion criteria: (1) Male, ages ≥ 50years; (2)Diagnosis of osteoporosis according to World Health Organization (WHO) criteria [19].

Exclusion criteria: (1) Long-term use of drugs affecting bone metabolism (e.g., glucocorticoids, bisphosphonates, active vitamin D); (2) History of primary bone tumors or bone metastases; (3) Previous treatment for osteoporosis; (4) Endocrine disorders (e.g., hypogonadism, hyperthyroidism, hyperparathyroidism); (5) Chronic diseases (e.g., chronic kidney disease, chronic liver disease, metabolic syndrome, and diabetes).

### BMD measurement and group division

BMD was measured using a Hologic Discovery Wi DXA system (Hologic Inc., Marlborough, MA, USA). The system underwent daily calibration and quality control (QC) with a manufacturer-provided phantom to ensure accuracy.For T-score calculation, we used the Hologic reference database for men. All BMD measurements were performed by experienced radiologic technologists who were blinded to the patients’ clinical information. The lowest T-score from the lumbar spine was used to classify patients into three groups: NO (normal): T-score > -1.0;ON (osteopenia): -2.5 < T-score ≤ -1.0༛OP (osteoporosis): T-score ≤ -2.5.

All lumbar spine DXA images were visually inspected for vertebral fractures, osteophytes, and abdominal aortic calcification. Scans with significant artifacts that could affect BMD measurements were excluded from the analysis.

### Laboratory measurements and AIP calculation

Serum N-MID osteocalcin (bone Gla protein, BGP) was quantified using the Roche e601 N-MID osteocalcin assay, with an intra- and inter-assay coefficient of variation (CV) of 4.49% to ensure precision. All measurements were conducted under standardized conditions to minimize variation.

Serum lipid profiles, including TC, TG, HDL, and LDL, were measured using standard enzymatic methods on the Beckman AU5800. Calibration was performed every two weeks using certified reference materials to ensure accuracy. All lipid parameters were expressed in mmol/L.

Participants fasted for at least 12 h before blood collection to standardize measurements and reduce variability. Samples were collected in the morning to minimize diurnal variations, and analyses were completed within 2 h.

**Fig. 1:**
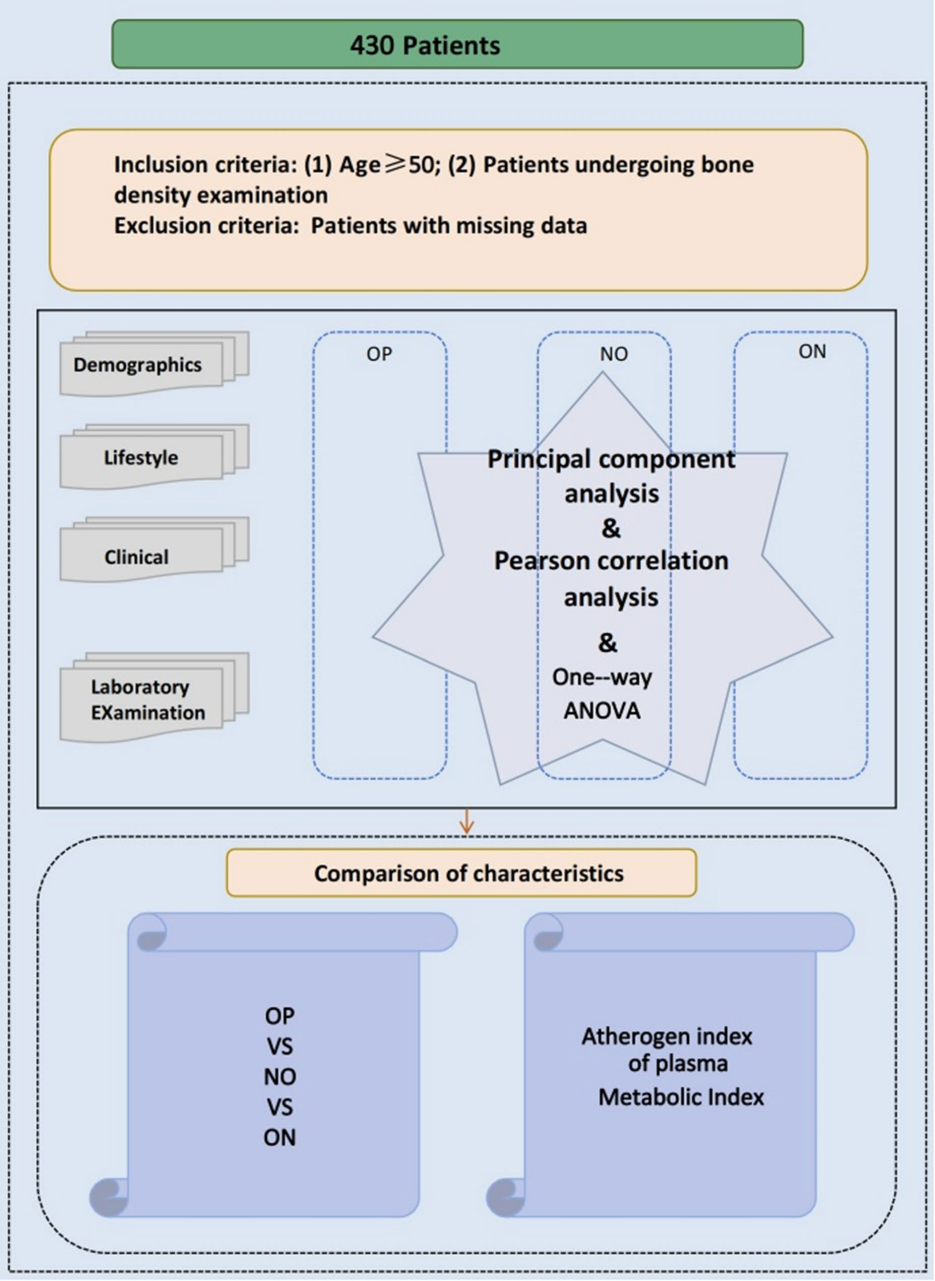
Flow chart

AIP calculation is described in the Introduction section. The study design and data analysis workflow are summarized in Fig. 1.

### Statistical analysis

The normality of continuous variables was assessed using the Shapiro–Wilk test, and the homogeneity of variances was evaluated using Levene’s test. Variables with a normal distribution were expressed as mean ± standard deviation (SD), while those with a non-normal distribution were presented as median (interquartile range, IQR). Categorical variables were summarized as frequencies and percentages.

Between-group differences were analyzed using ANOVA, followed by the Bonferroni post hoc test for multiple comparisons. Post-hoc Bonferroni comparisons of clinical and biochemical parameters among the three groups are presented in Table S1. Nonparametric tests (such as the Kruskal–Wallis test) were applied for variables that did not meet the assumptions of normality or homogeneity of variances.

Regression Analysis: To assess the relationship between AIP and BMD, multivariate linear regression analyses were performed (Table S2). Model 1 was unadjusted; Model 2 adjusted for age and Body Mass Index (BMI); and Model 3 further adjusted for platelet count, systolic blood pressure (SBP), diastolic blood pressure (DBP), 25-hydroxyvitamin D₃ (25(OH)D), N-MID osteocalcin, β-Collagen Degradation Products (β-CTX), and Procollagen Type I N-Terminal Propeptide (PINP). Regression coefficients (B), standard errors (SE), and standardized β coefficients (β) were reported, with stepwise regression used to evaluate independent associations between variables.

Ordinal Logistic Regression: Additionally, ordinal logistic regression was used to evaluate the association between AIP and BMD categories (normal, osteopenia, osteoporosis). Variables included in the model were AIP, age, BMI, platelet count, SBP, DBP, 25(OH)D, N-MID osteocalcin, β-CTX, and PINP (Table S3). Estimated coefficients (B), standard errors (SE), Wald statistics, p-values, and odds ratios (Exp(B)) were reported.

PCA was conducted to identify the most influential lipid-related variables associated with BMD. PCA was performed using varimax orthogonal rotation to facilitate the interpretation of component loadings. Components with eigenvalues greater than 1 (PC1 and PC2) were retained for further analysis. As PCA is an exploratory multivariate technique that does not involve hypothesis testing of pairwise correlations, correlation coefficients (r), p-values, or 95% confidence intervals (CIs) were not provided, as these are not typically reported in this type of analysis.

Correlation Analysis: Pearson correlation analysis was employed to examine associations between AIP, lipid parameters, and BMD (Fig. 1). Table 2 presents the correlation between the Atherogenic Index and male osteoporosis. All statistical tests were two-tailed, with a p-value of < 0.05 considered statistically significant.

Receiver operating characteristic (ROC) curve analysis was performed to assess the diagnostic performance of various variables in distinguishing different BMD status groups (normal, osteopenia, and osteoporosis). Figure S1 shows the ROC curves, and Table S4 provides a summary of the ROC analysis, including the area under the curve(AUC), 95% confidence intervals (CIs), cut-off values, sensitivity, and specificity. The AUC and 95% confidence intervals (CIs) were calculated and reported to evaluate the discriminative ability of each variable.

Although a formal power analysis was not conducted, the total sample size of 430 participants was deemed adequate based on previous studies with similar designs and effect sizes, ensuring sufficient statistical power to detect moderate group differences.

## Results

### General characteristics

A total of 430 male participants were included: 218 in the NO group, 133 in the ON group, and 79 in the OP group. The mean ages were comparable among the groups (NO: 63.62 ± 8.16; ON: 63.02 ± 7.32; OP: 63.61 ± 8.28 years). Significant between-group differences were observed in BMI, platelet count, alanine aminotransferase (ALT)(*P* = 0.003), Indirect bilirubin (IBIL)(*P* = 0.007), alkaline phosphatase (ALP)(*P* = 0.016), HDL (*P* = 0.000), β-CTX (*P* = 0.025), and N-MID osteocalcin (*p* = 0.015). Other baseline parameters, including age, SBP, DBP, pulse rate, lymphocyte count, neutrophil count, aspartate aminotransferase (AST), total protein, globulin, albumin, TBIL, DBIL (Direct Bilirubin), TC, LDL, serum calcium (Ca), serum magnesium (Mg), urea, uric acid (UA), serum phosphorus (P), 25(OH)D; PINP and parathyroid hormone (PTH) did not differ significantly(Table 1).These baseline differences, particularly in BMI and ALT levels, may reflect overall health status and potentially influence bone metabolism and bone health.Post-hoc Bonferroni tests revealed significant differences among the three groups for weight, height, BMI, platelet count, AST, ALP, AIP, HDL, and N-MID osteocalcin (*p* < 0.05). Full post-hoc comparison results are provided in Supplementary Table S1.

**Table 1:** Clinical and laboratory characteristics of participants stratified by bone mineral density groups

	Total	Normal	Osteopenia	Osteoporosis	*P*
N	430	218	133	79	
AIP	0.11 ± 0.29	0.14 ± 0.28	0.11 ± 0.29	0.03 ± 0.30	0.016
Age, Years	63.43 ± 7.92	63.62 ± 8.16	63.02 ± 7.32	63.61 ± 8.28	0.768
BMI (kg/m^2^)	24.26 ± 3.29	25.25 ± 3.05	23.74 ± 2.73	22.43 ± 3.80	0.000
SBP(mmHg)	131.77 ± 17.15	133.06 ± 16.49	129.83 ± 17.7	131.49 ± 17.9	0.229
DBP(mmHg)	81.05 ± 11.25	81.57 ± 10.92	80.08 ± 12.01	81.25 ± 10.85	0.480
Pulse	80.72 ± 11.28	81.06 ± 12.12	80.85 ± 10.21	79.56 ± 10.62	0.590
Platelet Count(×10⁹/L)	208.96 ± 67.23	213.82 ± 64.85	191.27 ± 63.66	225.11 ± 73.71	0.001
Lymphocyte Count(×10⁹/L)	2.17 ± 6.91	2.44 ± 9.58	1.75 ± 0.67	2.12 ± 2.4	0.662
Neutrophil Count(×10⁹/L)	3.89 ± 2.0	3.84 ± 1.51	3.84 ± 2.63	4.14 ± 2.0	0.225
ALT(U/L)	21.66 ± 13.15	20.81 ± 12.67	21.78 ± 12.38	23.78 ± 15.45	0.003
AST(U/L)	21.36 ± 11.12	20.14 ± 8.82	21.12 ± 9.45	25.11 ± 17.14	0.875
Total Protein(g/L)	65.14 ± 6.15	65.23 ± 6.57	65.18 ± 5.87	64.82 ± 5.40	0.534
Albumin(g/L)	39.97 ± 4.4	39.99 ± 4.46	40.2 ± 4.37	39.5 ± 4.29	0.437
Globulin(g/L)	25.32 ± 4.29	25.56 ± 4.59	24.96 ± 3.75	25.27 ± 4.32	0.203
TBIL(µmol/L)	15.24 ± 6.93	14.98 ± 6.83	16.1 ± 7.78	14.52 ± 6.93	0.157
DBIL(µmol/L)	2.92 ± 2.0	2.79 ± 1.92	3.2 ± 2.4	2.82 ± 1.32	0.261
IBIL(µmol/L)	12.29 ± 5.5	12.14 ± 5.48	12.89 ± 6.0	11.69 ± 4.61	0.007
ALP(U/L)	0.10 ± 0.29	0.14 ± 0.28	0.11 ± 0.29	0.03 ± 0.30	0.016
TC(mmol/L)	4.27 ± 1.22	4.31 ± 1.27	4.15 ± 1.14	4.39 ± 1.21	0.341
TG(mmol/L)	1.67 ± 1.45	1.74 ± 1.64	1.60 ± 1.14	1.56 ± 1.39	0.520
HDL(mmol/L)	1.10 ± 0.27	1.08 ± 0.27	1.07 ± 0.26	1.22 ± 0.28	0.000
LDL(mmol/L)	2.65 ± 0.82	2.79 ± 0.83	2.57 ± 0.75	2.66 ± 0.89	0.375
Urea(mmol/L)	6.62 ± 5.39	7.10 ± 7.05	6.43 ± 3.13	5.59 ± 1.70	0.091
Cr(µmol/L)	76.46 ± 60.35	78.0 ± 40.0	79.25 ± 94.84	67.5 ± 16.27	0.339
UA(µmol/L)	333.50 ± 91.43	335.53 ± 95.53	337.62 ± 85.14	320.98 ± 88.94	0.396
Ca(mmol/L)	2.26 ± 0.19	2.26 ± 0.22	2.26 ± 0.11	2.27 ± 0.17	0.820
P(mmol/L)	1.19 ± 0.23	1.21 ± 0.25	1.17 ± 0.21	1.16 ± 0.19	0.169
Mg(mmol/L)	0.86 ± 0.20	0.87 ± 0.21	0.88 ± 0.24	0.84 ± 0.07	0.550
25(OH)D(ng /mL)	47.67 ± 21.81	47.49 ± 20.97	48.42 ± 22.76	46.92 ± 22.67	0.876
N-MID osteocalcin (ng /mL)	20.15 ± 52.44	14.66 ± 19.85	20.61 ± 16.77	16.89 ± 14.26	0.015
PTH(p g /mL)	39.74 ± 53.26	43.61 ± 69.71	34.09 ± 28.57	38.57 ± 24.57	0.261
PINP(ng /mL)	49.08 ± 60.43	49.43 ± 77.69	46.63 ± 35.95	52.25 ± 32.91	0.802
β-CTX(ng /mL)	488.85 ± 433.53	433.7 ± 460.23	535.28 ± 419.03	562.87 ± 359.95	0.025

Abbreviation: BMI: Body Mass Index; SBP: systolic blood pressure; DBP: diastolic blood pressure; ALT: alanine aminotransferase; AST: aspartate aminotransferase; TBIL: total bilirubin; DBIL: direct bilirubin; IBIL: indirect bilirubin; ALP: alkaline phosphatase; TC: Total Cholesterol; TG: triglycerides; HDL: high-density lipoprotein cholesterol; LDL: Low-density lipoproteincholesterol; Cr: Creatinine; UA: uricacid; Ca: serumcalcium; P:serumphosphorus; Mg: serum magnesium;25(OH)D:25 hydroxyvitamin D_3_;N-MID osteocalcin: a stable fragment of osteocalcin (bone Gla protein, BGP); PTH: parathyroid hormone; PINP: Procollagen I N-TerminalPropeptide;β-CTX:β-Collagen Degradation Products. Values are presented as mean ± SD. Normal reference: ALT:0 ~ 40U/L; AST:0 ~ 40U/L; TBIL3.4 ~ 20µmol/L; DBIL:0 ~ 6.8µmol/L; IBIL:2.6–13.2µmol/L; ALP40-50U/L; a;TC:<5.0mmol/L; TG:<1.7mmol/L; HDL:≥1.0mmol/L; LDL:<2.5mmol/L; Cr:60 ~ 118µmol/L; UA:149 ~ 416µmol/L; Ca:2.1 ~ 2.6mmol/L; P:0.8 ~ 1.5mmol/L; Mg:0.7 ~ 1.1mmol/L;25(OH)D₃:≥ 30 ng/mL; N-MID osteocalcin:10–30 ng/mL; PTH:10–65 pg/mL; PINP and β-CTX: No internationally standardized reference range has been established

### Principal component analysis

PCA was applied to the five lipid-related variables (AIP, TC, TG, HDL and LDL) to reduce multicollinearity and identify independent lipid patterns associated with BMD. The analysis employed a varimax orthogonal rotation to enhance interpretability.

**Fig. 2:**
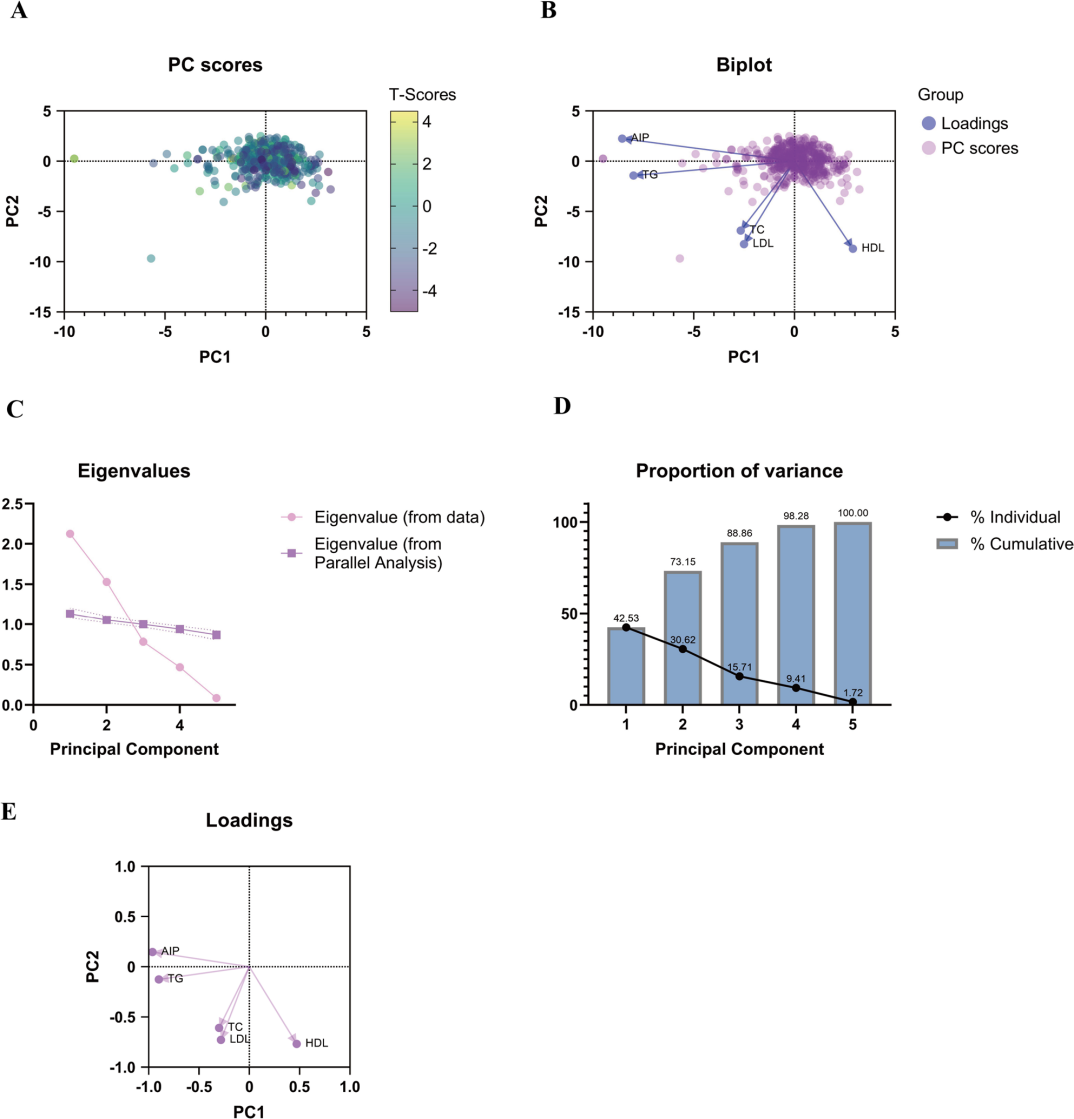
Principal component analysis of lipid-related variables. (**A**) PC score plot of individual patients in the PC1–PC2 space, colored by BMD T-scores. (**B**) Biplot showing PC scores and variable loadings for AIP, TG, TC, LDL, and HDL on PC1 and PC2. (**C**) Scree plot of eigenvalues from the data and from parallel analysis. (**D**) Proportion of variance explained by each principal component and the cumulative variance. (**E**) Loading plot of AIP, TG, TC, LDL, and HDL on PC1 and PC2.AIP: atherogenic index of plasma; TG: triglycerides; TC: Total Cholesterol; HDL: high-density lipoprotein cholesterol

Two principal components with eigenvalues greater than 1 were extracted according to the Kaiser criterion: PC1 (eigenvalue = 2.127, variance contribution = 42.53%) and PC2 (eigenvalue = 1.531, variance contribution = 30.62%), together explaining 73.15% of the total variance. Among these variables, AIP and TC showed the highest loadings on PC1, indicating their dominant contribution to the lipid pattern related to BMD. The scree plot confirmed that the first two components accounted for most of the explained variance, and the score plots demonstrated a clear separation trend among groups along PC1 (Fig. 2).

### Correlation between atherogenic index and male osteoporosis

Pearson correlation analysis demonstrated significant associations between AIP (*p* = 0.003), TG (*p* = 0.047), and HDL (*p* < 0.001) with the lowest BMD T-score, whereas TC (*p* = 0.418) and LDL (*p* = 0.373) showed no significant correlations (Table 2). Significance was denoted by “*” for *p* < 0.05 and “ns” for non-significant results. Interestingly, the lack of association for TC and LDL is clinically relevant, as previous studies have suggested potential links between lipid metabolism and bone health. Our findings indicate that TC/LDL levels may not serve as reliable indicators of osteoporosis risk in men, underscoring the greater importance of parameters such as AIP and HDL in reflecting lipid-related mechanisms of bone loss.

**Table 2:** Correlation between atherogenic index and male Osteoporosis

Atherogenic index	Correlation coefficient	95%CI	*P*
AIP	0.133	0.039, 0.225	0.003(*)
TC	0.010	-0.085, 0.104	0.418(ns)
TG	0.081	-0.014, 0.174	0.047(*)
HDL	-0.159	-0.250, -0.065	0.000(*)
LDL	-0.016	-0.079, 0.110	0.373(ns)

Abbreviation: AIP: atherogenic index of plasma; TC: Total Cholesterol; TG: triglycerides; HDL: high-density lipoprotein cholesterol; LDL: Low-density lipoprotein cholesterol; CI: Confidence Interval

Additionally, BMI was positively correlated with weight (*r* = 0.86, *p* < 0.001) and negatively correlated with HDL (*r* = − 0.21, *p* < 0.05). Platelet count showed a moderate positive correlation with AST (*r* = 0.34, *p* < 0.05), suggesting potential metabolic and hepatic interactions influencing bone health.

### Regression analysis results

To further explore the relationships between lipid markers and bone mineral density, we performed multivariate linear regression analysis (Table S2). In Model 1 (unadjusted), AIP was significantly associated with the lowest T-score (B = 0.756, *p* = 0.006). However, in Model 2, after adjusting for age and BMI, AIP’s effect diminished (B = 0.276, *p* = 0.323), and further adjustment for additional variables in Model 3 showed no significant association (B = 0.280, *p* = 0.325). These results indicate that while AIP may be related to BMD in an unadjusted model, the relationship weakens after accounting for confounding factors such as age and BMI.

Additionally, ordinal logistic regression analysis (Table S3) was conducted to assess the association of AIP with different BMD categories. AIP did not show a significant relationship with BMD categories (Exp(B) = 0.856, *p* = 0.670), suggesting that other variables, particularly BMI, may be more predictive of BMD classification.

### Between-group comparison

**Fig. 3:**
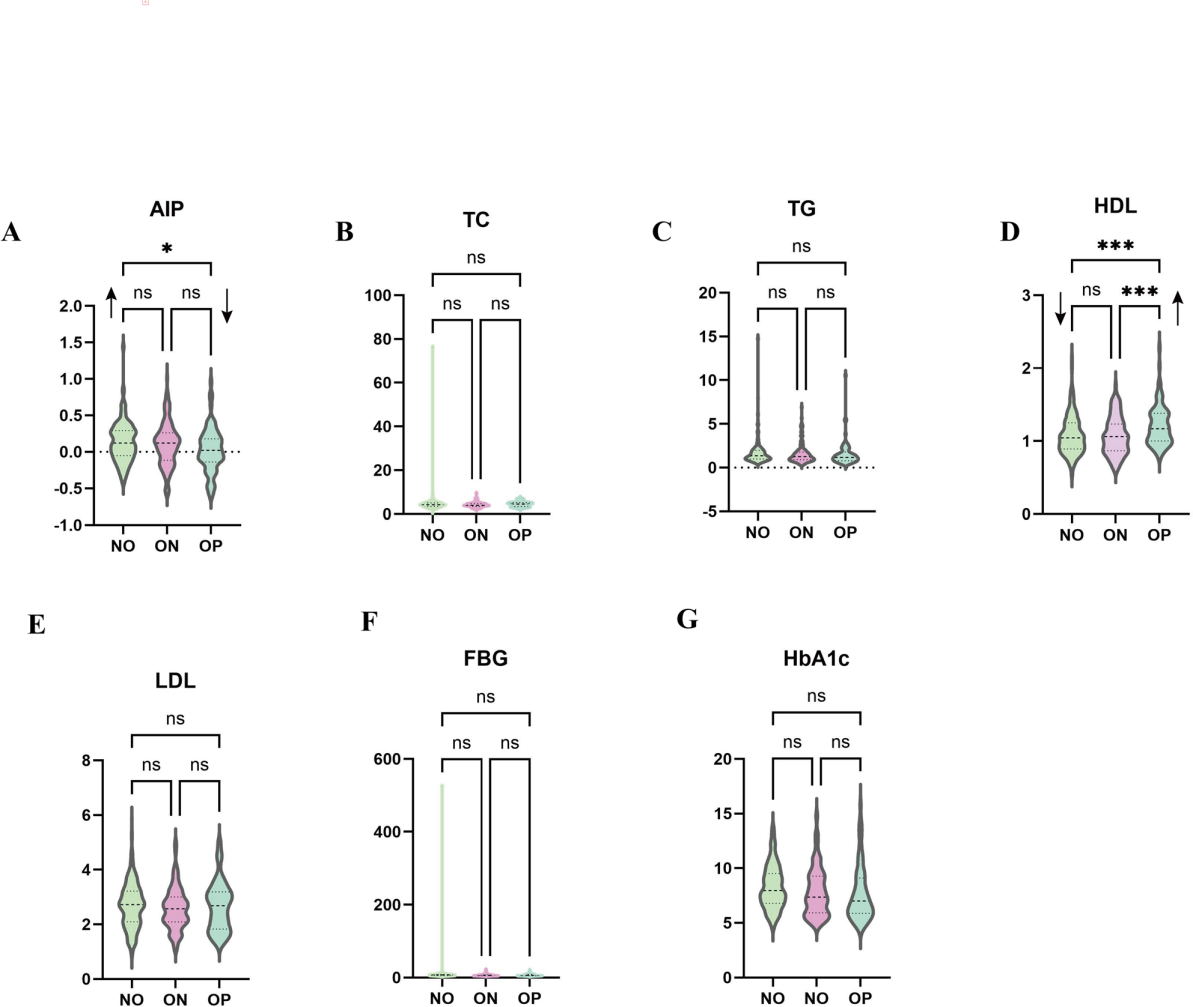
Violin plots of lipid and glycemic variables according to BMD status. Violin plots show the distribution of (**A**) atherogenic index of plasma (AIP), (**B**) total cholesterol (TC), (**C**) triglycerides (TG), (**D**) high-density lipoprotein cholesterol (HDL), (**E**) low-density lipoprotein cholesterol (LDL), (**F**) fasting blood glucose (FBG), and (**G**) glycated hemoglobin (HbA1c) across the normal (NO), osteopenia (ON), and osteoporosis (OP) groups. Dashed lines within each violin indicate the median and interquartile range. Group comparisons were performed using post-hoc tests, with significance annotated as *P < 0.05, ***P < 0.001, and ns = not significant. The OP group shows significantly higher AIP and HDL compared with the NO group, whereas TC, TG, LDL, FBG, and HbA1c do not differ significantly among groups

Based on the PCA and correlation findings, one-way ANOVA was performed to further assess differences in lipid-related markers across the three BMD groups. The analysis revealed:

AIP: significantly higher in the OP group compared with the NO group (*p* = 0.013), but no significant differences between ON and the other groups.HDL: significantly higher in the OP group compared with both NO and ON groups (*p* < 0.001).TC, TG, and LDL: no significant differences among the three groups (all *p* > 0.05)(Fig. 3). Consistent with the correlation analysis, the absence of group differences in TC and LDL further supports the interpretation that these conventional lipid markers may have limited relevance in male osteoporosis, in contrast to the stronger and more consistent associations observed for AIP and HDL(Fig. 3).

### ROC curve analysis

ROC analysis was conducted to evaluate the diagnostic performance of various lipid-related parameters. Figure S1 illustrates the ROC curves for AIP, HDL, and other lipid parameters, while Table S4 provides a detailed summary of the ROC analysis, including AUC, 95% confidence intervals (CIs), cut-off values, sensitivity, and specificity. The AUC values indicate the discriminative ability of each variable in distinguishing different BMD status groups.

## Discussion

### Research background and summary of results

Compared to postmenopausal osteoporosis, male osteoporosis remains largely underdiagnosed and undertreated due to lower screening frequency and controversies surrounding BMD testing standards [20, 21]. Our study aims to explore the role of atherosclerosis-related biomarkers in male osteoporosis. We found that biomarkers related to atherosclerosis are significantly associated with decreased BMD, suggesting they may be involved in the pathophysiological processes of male osteoporosis. The economic burden of osteoporosis is significant, with fractures costing €37.5 billion in Europe in 2017, and projected to rise to €47.4 billion by 2030, particularly due to hip fractures, which account for the largest share of costs (57%) [22].

### Association between AIP and osteoporosis

Our findings align with previous studies indicating a non-linear association between AIP and BMD, with sex and age potentially modifying this relationship [23, 24]. While HDL-BMD associations remain inconsistent—sometimes positive, negative, or U-shaped, especially among males [25]—research specific to men is limited. This highlights the need for further studies to validate these findings. Our study provides novel evidence linking atherosclerosis-related biomarkers, particularly AIP, to male osteoporosis, a topic previously focused primarily on postmenopausal women [16].

### Mechanism discussion

Consistent with our hypothesis, AIP was significantly higher in the osteoporosis group than in the normal BMD group, suggesting that lipid-related markers may contribute to the pathophysiological processes underlying bone loss in men [26–30]. Notably, principal component analysis revealed that AIP and TC were the most influential lipid variables, indicating they may serve as more comprehensive risk assessment tools than individual lipid markers [31].

Dyslipidemia may affect bone metabolism through inflammation–oxidative stress pathways that enhance osteoclastogenesis and impair osteoblast function. HDL provides antioxidative and anti-inflammatory protection, while oxidized LDL promotes RANKL(Receptor Activator of Nuclear Factor-κB Ligand) expression and bone resorption, further contributing to bone loss. This interpretation aligns with evidence linking lipid toxicity, marrow adiposity, and Wnt(Wingless/Integrated signaling pathway)/PPARγ(Peroxisome Proliferator-Activated Receptor Gamma) signaling to skeletal health [32].

In addition to lipid-related mechanisms, recent genetic studies suggest that specific gene polymorphisms may influence osteoporosis susceptibility through regulation of osteoblast and osteoclast differentiation. For instance, the osteoprotegerin (OPG) G209A variant was found to be significantly associated with osteopenia and osteoporosis risk in the Turkish population, implicating the RANKL–RANK(Receptor Activator of Nuclear Factor-κB)–OPG signaling axis as a key genetic pathway mediating bone fragility [33].

Correlation analysis further supported this conclusion, showing significant associations between AIP, TG, and HDL with the lowest BMD T-score, whereas TC and LDL did not exhibit such relationships, which is consistent with some research findings [34, 35].This pattern suggests that specific lipid subcomponents, particularly HDL, may have a stronger regulatory effect on bone metabolism than total cholesterol. Interestingly, HDL was negatively correlated with BMD, indicating that under certain metabolic conditions, HDL, despite its cardiovascular protective role, could have an adverse impact on bone mass. This paradox contrasts with some epidemiological findings [25], possibly due to differences in population characteristics, metabolic status, or hormonal influences.

Although principal component analysis identified AIP as one of the dominant lipid-related contributors to BMD variation, its independent predictive effect on BMD categories was not significant in the multivariate and ordinal logistic regression models after adjusting for age, BMI, and metabolic factors. This apparent inconsistency arises from the methodological differences between unsupervised and supervised analyses. PCA evaluates the structural contribution of variables to total variance, whereas regression models assess independent predictive strength after accounting for covariates. Therefore, AIP may act as an integrative marker reflecting overall dyslipidemic status rather than an independent determinant of bone mineral density. This interpretation aligns with the notion that lipid abnormalities exert indirect effects on bone metabolism via intermediary factors such as BMI, inflammation, and oxidative stress.

Taken together, these statistical findings indicate that while AIP contributes substantially to the overall lipid–BMD structure, its direct effect becomes attenuated after accounting for metabolic confounders.Between-group comparisons revealed significant differences in AIP and HDL levels between the normal and osteoporosis groups, whereas TG, TC, and LDL showed no significant differences. This selective association suggests that male bone health may be influenced more by qualitative alterations in lipid profiles than by overall lipid levels. AIP, which reflects the balance between TG and HDL, may indicate a lipid pattern associated with atherosclerosis [36], Through shared mechanisms involving inflammation and oxidative stress, this pattern could also contribute to enhanced bone resorption [37].

Our multivariate regression analysis showed that the effect of AIP on BMD diminished after adjusting for age, BMI, and other metabolic factors, indicating potential interactions between AIP and these variables. The initial correlation between AIP and the lowest T-score was no longer significant after adjusting for confounders (Table S2).

### Clinical significance and limitations

This study has several limitations. First, its retrospective design precludes causal inference, and longitudinal studies are needed to clarify whether lipid abnormalities precede bone loss or arise as a consequence of osteoporosis-related metabolic alterations. Second, bone mineral density was assessed only at the lumbar spine, without including other clinically relevant skeletal sites such as the femoral neck, total hip, or distal radius, which are recommended by international guidelines for accurate diagnosis [38]. Third, the sample size of 430 patients is relatively small compared to large-scale studies, limiting statistical power and generalizability. Finally, this was a single-center study conducted in hospitalized male patients at Shanxi Medical University First Hospital, lacking external validation across multicenter, multi-ethnic, and larger population-based cohorts. Additionally, data on lipid-lowering therapies (e.g., statins) were not available, potentially introducing confounding as statins can influence both lipid profiles and bone metabolism.

A limitation of the current study is the lack of data on secondary causes of osteoporosis and key confounders such as smoking, alcohol use, and medications. These factors could influence bone metabolism and lipid profiles, and their absence may impact the interpretation of our results. Future studies should incorporate these variables to refine the relationship between lipid metabolism and bone health in men.Additionally, hypogonadism is a leading cause of male osteoporosis and is known to correlate with lipid metabolism and AIP levels. However, due to the absence of testosterone, SHBG(Sex hormone–binding globulin), and LH/FSH (Luteinizing hormone/Follicle-stimulating hormone)measurements in our study, we acknowledge that hypogonadism may act as a confounding factor or mediator in the relationship between lipid markers and bone mineral density. We have highlighted this limitation and suggested the need for future studies to incorporate these data.

Despite these limitations, our study has notable strengths. It addresses male osteoporosis, a condition that remains underdiagnosed and underexplored compared to postmenopausal osteoporosis. By integrating multiple lipid parameters and using principal component analysis, we identified AIP and TC as dominant contributors to bone health variation. This approach provides novel insights into the lipid–bone relationship from a sex-specific perspective and offers clinical implications for early screening and preventive strategies.

### Future research directions

In conclusion, this study demonstrates that AIP and HDL are significantly associated with male osteoporosis, whereas TC and LDL show no clear relationship. Taken together, our findings provide novel evidence linking atherosclerosis-related lipid patterns to bone metabolism in men, highlighting the potential of AIP as an integrated biomarker for early risk stratification and intervention.

These findings suggest that AIP may not only serve as a marker of cardiovascular risk but also have potential relevance to bone health in men.As this was a cross-sectional retrospective study, the observed associations do not establish temporality or causality; therefore, multicenter, large-scale prospective studies are warranted to confirm these findings and further elucidate the mechanisms linking lipid metabolism to bone health.Given the substantial socioeconomic burden of osteoporosis, early identification of high-risk individuals through routine lipid profiling—particularly incorporating AIP—may represent a simple and cost-effective preventive strategy.

## Supplementary Information

Below is the link to the electronic supplementary material.

Supplementary Material 1

## Data Availability

The datasets used and analyzed during the current study are available from the corresponding author on reasonable request.

## Abbreviations

AIP: Atherogenic index of plasma
BMD: Bone mineral density
BMI: Body Mass Index
ANOVA: Analysis of Variance
SBP: Systolic blood pressure
DBP: Diastolic blood pressure
ALT: Alanine aminotransferase
AST: Aspartate aminotransferase
TBIL: Total bilirubin
DBIL: Direct bilirubin
IBIL: Indirect bilirubin
ALP: Alkaline phosphatase
TC: Total Cholesterol
TG: Triglycerides
HDL: High-density lipoprotein cholesterol
LDL: Low-density lipoprotein cholesterol
Cr: Creatinine
UA: Uric acid
Ca: Serum calcium
P: Serum phosphorus
Mg: Serum magnesium
25(OH)D: 25 hydroxyvitamin D_3_
N-MID osteocalcin: N-terminal and mid-region osteocalcin
PTH: Parathyroid hormone
PINP: Procollagen I N-Terminal Propeptide
β-CTX: β-Collagen Degradation Products
RANKL: Receptor Activator of Nuclear Factor-κB Ligand
Wnt: Wingless/Integrated signaling pathway)/PPARγ(Peroxisome Proliferator-Activated Receptor Gamma
SHBG: Sex hormone–binding globulin
LH/FSH: Luteinizing hormone/Follicle-stimulating hormone
