# Supplementary material for: Novel association between atherogenic index of plasma and bone mineral density in men: a retrospective analysis
Source: BMC Endocr Disord. 2026 Jan 6;26:33. doi: 10.1186/s12902-025-02128-3 (PMC12870077; doi:10.1186/s12902-025-02128-3)
Supplement: Supplementary file 1 — Supplementary Material 1 [file 12902_2025_2128_MOESM1_ESM.docx]

*Title Page*

**Association of Atherogenic Index of Plasma and Lipid Profiles with Bone Mineral Density in Male Osteoporosis**

Bing Liu^2#^,Yue Liu^3^,Qing Xue^3^ ,Fei Gao^1*^,Hao Qi ^1*^

TABLE S1 Post-hoc Bonferroni comparisons of clinical and biochemical parameters among the three groups.

TABLE S2 Multivariate linear regression analysis for the lowest T-score (Model 1–3)

**TABLE S3 Ordinal logistic regression for BMD categories**

TABLE S4 ROC analysis summary (AUC, 95% CI, cut-off, sensitivity, specificity)

FIGURE S1 ROC curves

**TABLE S1 Post-hoc Bonferroni comparisons of clinical and biochemical parameters among the three groups.**

| Parameter | Significant Comparison (Group 0–2) | Mean Difference(0–2) | p-value | Direction |
| --- | --- | --- | --- | --- |
| BMI(kg/m^2^ ) | 0–1, 0–2, 1–2 | 1.51, 2.82, 1.30 | <0.05 | 0 > 1 > 2 |
| Platelet Count(×10⁹/L) | 0–1, 1–2 | 22.55, 33.84 | <0.01 | 1 < 0, 2 |
| AST(U/L) | 0–2, 1–2 | 4.97, 3.99 | <0.05 | 2 > 0, 1 |
| ALP(U/L) | 0–1, 0–2 | 8.31, 9.58 | <0.05 | 1, 2 > 0 |
| HDL(mmol/L) | 0–2, 1–2 | 0.137, 0.145 | <0.001 | 2 > 0, 1 |
| N-MID osteocalcin(ng  /mL) | 0–2 | 19.89 | 0.011 | 2 > 0 |

There were no significant differences among the three groups (after Bonferroni correction) in the following variables: age, systolic blood pressure, diastolic blood pressure, pulse, lymphocyte count, neutrophil count, alanine aminotransferase (ALT), total protein, albumin, globulin, total bilirubin, direct bilirubin, indirect bilirubin, total cholesterol, triglycerides, LDL, urea, creatinine, uric acid, calcium, phosphorus, magnesium, 20(OH)VD, parathyroid hormone, PINP, β-CTX, fasting blood glucose (FBG), and HbA1c.
Among them, some pairwise comparisons showed marginal significance (e.g., β-CTX and HbA1c with P ≈ 0.07–0.16), but these did not reach the threshold after Bonferroni correction.

**Table S2 Multivariate linear regression analysis for the lowest T-score (Model 1–3)**

| **Variables** | **Model 1** B (SE) β p value | **Model 2** B (SE) β p value | **Model 1** B (SE) β p value |
| --- | --- | --- | --- |
| AIP | 0.756 (0.273) 0.133 0.006 | 0.276 (0.278) 0.048 0.323 | 0.280 (0.284) 0.049 0.325 |
| Age，years | - | 0.019 (0.010) 0.092 0.048 | 0.012 (0.011) 0.056 0.282 |
| BMI(kg/m^2^ ) | - | 0.169 (0.024) 0.336 <0.001 | 0.164 (0.024) 0.325 <0.001 |
| Platelet Count(×10⁹/L) | - | - | 0.000 (0.001) –0.009 0.842 |
| SBP(mmHg) | - | - | 0.006 (0.006) 0.058 0.348 |
| DBP(mmHg) | - | - | –0.007 (0.009) –0.048 0.435 |
| 25(OH)D(ng  /mL) | - | - | 0.004 (0.004) 0.057 0.220 |
| N-MID osteocalcin(ng  /mL) | - | - | –0.012 (0.006) –0.105 0.052 |
| β-CTX(ng  /mL) | - | - | 0.000 (0.000) –0.089 0.275 |
| PINP(ng  /mL) | - | - | 0.001 (0.002) 0.042 0.564 |
| R² / Adjusted R² | 0.018 / 0.016 | 0.147 / 0.141 | 0.156 / 0.136 |
| F (p-value) | 7.67 (**p = 0.006**) | 8.09 (**p < 0.001**) | 7.71 (**p < 0.001)** |

Model 1 unadjusted; Model 2 adjusted for age and BMI; Model 3 further adjusted for platelet count, SBP, DBP, 25(OH)D, N-MID osteocalcin:, β-CTX and PINP. Bold values indicate p < 0.05.

| **Variables** | **Estimate(B)** | **SE** | **Wald** | **df** | **p value** | **Exp(B)** | **95% CI for Exp(B）** |
| --- | --- | --- | --- | --- | --- | --- | --- |
| AIP | 0.155 | 0.363 | 0.182 | 1 | 0.670 | 0.856 | [0.422, 1.831] |
| Age,years | -0.004 | 0.014 | 0.068 | 1 | 0.795 | 0.996 | [0.975, 1.017] |
| BMI(kg/m^2^ ) | -0.224 | 0.035 | 41.171 | 1 | 0.000 | 0.799 | [0.746, 0.855] |
| Platelet Count(×10⁹/L) | -0.001 | 0.001 | 0.215 | 1 | 0.643 | 0.999 | [0.997, 1.001] |
| SBP(mmHg) | -0.004 | 0.008 | 0.274 | 1 | 0.601 | 0.996 | [0.981, 1.010] |
| DBP(mmHg) | 0.005 | 0.012 | 0.167 | 1 | 0.683 | 1.005 | [0.970, 1.041] |
| 25(OH)D(ng/mL) | -0.001 | 0.005 | 0.085 | 1 | 0.771 | 0.999 | [0.990, 1.010] |
| BGP(ng  /mL) | 0.030 | 0.007 | 17.005 | 1 | 0.000 | 1.031 | [1.016, 1.044] |
| β-CTX(ng  /mL) | 0.001 | 0.000 | 7.268 | 1 | 0.007 | 1.001 | [1.000, 0.001] |
| PINP(ng  /mL) | -0.002 | 0.002 | 0.860 | 1 | 0.354 | 0.998 | [0.994, 1.002] |

**Table S3 Ordinal logistic regression for BMD categories**

**Estimate (B)** indicates the direction and strength of the relationship between the variable and the outcome. **Standard Error (SE)** reflects the precision of the estimate. **Wald** tests the significance of the variable; higher values indicate stronger significance. **df (degrees of freedom)** represents the number of independent pieces of information used in the estimation. **p-value (Sig.)** indicates statistical significance; values less than 0.05 are considered significant. **Exp(B)** is the odds ratio (OR), with values greater than 1 indicating a positive effect and values less than 1 indicating a negative effect.**95% Confidence Interval for Exp(B)** shows the range within which the true odds ratio likely lies. If the interval includes 1, the variable may not be significant.

**Table S4 ROC analysis summary (AUC, 95% CI, cut-off, sensitivity, specificity)**

| Variables | **AUC** | **Std. Error** | **Asymptotic Sig.** | **95% CI (Lower Bound)** | **95% CI (Upper Bound)** |
| --- | --- | --- | --- | --- | --- |
| BMI(kg/m^2^ ) | 0.324 | 0.035 | 0.000 | 0.255 | 0.393 |
| TG | 0.452 | 0.037 | 0.181 | 0.379 | 0.525 |
| HDL | 0.648 | 0.032 | 0.000 | 0.586 | 0.710 |
| 25(OH)D(ng/mL) | 0.482 | 0.038 | 0.615 | 0.408 | 0.556 |
| BGP(ng  /mL) | 0.567 | 0.037 | 0.063 | 0.494 | 0.640 |
| β-CTX(ng  /mL) | 0.0606 | 0.035 | 0.003 | 0.537 | 0.675 |
| AIP | 0.404 | 0.036 | 0.008 | 0.334 | 0.475 |

**AUC (Area Under the Curve)**: Represents the discriminatory power of each variable. Values closer to 1 indicate better performance, while values closer to 0.5 suggest poor discriminatory ability. **Standard Error (Std. Error)**: Measures the variability of the AUC estimate. A smaller standard error indicates a more precise estimate.**Asymptotic Sig. (p-value)**: The p-value associated with the null hypothesis that the true AUC equals 0.5. A value less than 0.05 typically indicates that the variable has statistically significant discriminatory ability. **95% Confidence Interval (CI)**: The range within which the true AUC is likely to fall with 95% confidence. If the interval includes 0.5, the discriminatory ability of the variable may not be statistically significant.

**Figure S1 ROC curves**

**
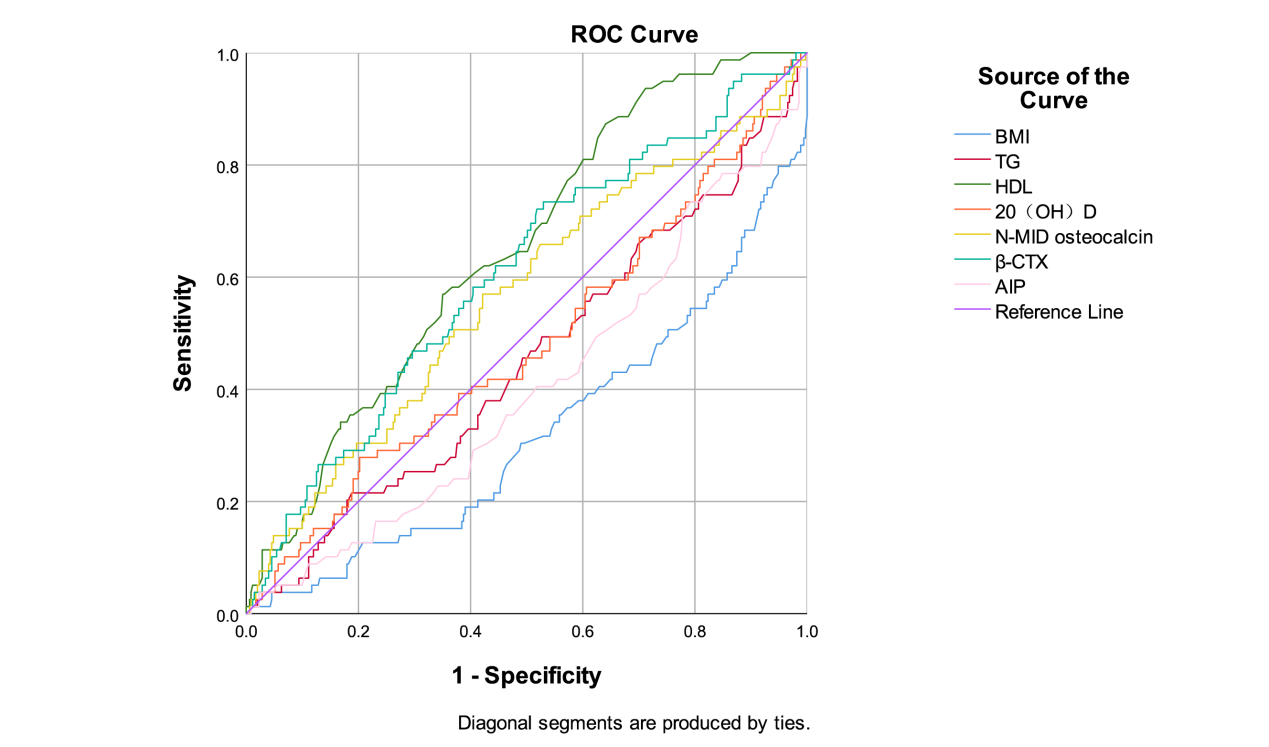
**
